# Using the Mothers Object Relations Scale for early childhood development research in rural India: Findings from the Early Life Stress Sub-study of the SPRING Cluster Randomised Controlled Trial (SPRING-ELS)

**DOI:** 10.12688/wellcomeopenres.16591.2

**Published:** 2022-01-31

**Authors:** Sunil S. Bhopal, Reetabrata Roy, Deepali Verma, Divya Kumar, Bushra Khan, Seyi Soremekun, John Oates, Gauri Divan, Betty R. Kirkwood

**Affiliations:** 1 Population Health Sciences Institute, Newcatle University, Newcastle upon Tyne, UK; 2 Maternal & Child Health Intervention Research Group, Department of Population Health, Faculty of Epidemiology & Population Health, London School of Hygiene & Tropical Medicine, London, UK; 3 Sangath, New Delhi, India; 4 Department of Psychology, University of Karachi, Karachi, Pakistan; 5 Department of Clinical Research, Faculty of Infectious & Tropical Diseases, London School of Hygiene & Tropical Medicine, London, UK; 6 Faculty of Wellbeing, Education & Language Studies, Open University, Milton Keynes, UK

**Keywords:** child, infant, epidemiology, child development, early childhood development, nurturing care, mother-child relations, object relations, attachment

## Abstract

**Background:** The World Health Organization and others promote responsive caregiving to support all children to thrive, particularly in low- and middle-income countries. The 14-item Mother’s Object Relations Scales – Short Form (MORS-SF) may be of use in research and public health programmes because of its basis in attachment theory and ability to capture parental feelings towards their child.

**Methods:** We culturally adapted the MORS-SF for use with mothers in the SPRING home visits trial when their infants were 12 months old. The same dyads were assessed using the HOME inventory concurrently and Bayley Scales of Infant Development III (BSID-III) at 18 months of age. Mixed effects linear regression was used to examine associations between MORS-SF (explanatory variable) and HOME-IT, and the cognitive, language and motor domains of BSID-III (outcome variables).

**Results: **1273 dyads completed all assessments. For the motor and language BSID-III scales and for HOME-IT there were strong and positive associations with the MORS-SF warmth sub-scale, and strong and negative associations with the invasion sub-scale. Important but less strong associations were seen with the BSID-III cognitive scale. Evidence of interaction suggested that both are individually important for child development.

**Conclusions:** This is the first time MORS-SF has been used in India where optimising responsive caregiving is of importance in supporting all children to reach their potential. It is also the first time that the tool has been used in relation to child development. MORS-SF could be a valuable addition to evaluation in early childhood development.

## Key messages

- Optimal early childhood development is crucial to ensuring all children have the opportunity to reach their potential, particularly in low- and middle-income countries where the burden is greatest.
- Measuring child development and related constructs is important for monitoring progress and evaluating programmes and research work.
- Mother’s Object Relations Scales – Short Form (MORS-SF) was suitable for use in rural India and is likely to be adaptable to other contexts.
- MORS-SF was strongly associated with HOME-IT scores at 12 months of age, and with Bayley Scales of Infant Development III (BSID-III) scores measured when the same infants were 18 months old.
- MORS-SF is a promising tool for use in early childhood development programmes in low- and middle-income countries.

## Introduction

The Lancet Series on early childhood development concluded that 250 million children under the age of five years who live in low- and middle-income countries (LMICs) are at high risk of not reaching their developmental potential ^1^. Momentum is now growing to improve this situation through improved nutrition, protection from threats, provision of learning opportunities, and caregiver interactions that are stimulating, responsive, and emotionally supportive.

The recently launched World Health Organization Nurturing Care Framework for Early Childhood Development has a focus on responsive caregiving as a way in which to create a world where all children have the opportunity to thrive ^2^. This sort of high-quality caregiver support is an important influence in mitigating risks to children’s development ^3^ with caring interactions supporting children to grow, learn and develop. Mothers do much of this care in young children. Mothers who are physically and mentally well, have adequate social support and are able to provide an age-appropriate and safe environment to their children are more likely to be able to care well for their children ^4^.

The Mother’s Object Relations Scales – Short Form (MORS-SF) was developed in the 1990s for assessing areas of difficulty in the early mother-infant relationship ^5^. It has a basis in attachment theory and attempts to measure an element of responsive-caregiving, both of which are foundational for optimal child development. The premise is that a mother’s perception of her infant influences the ways in which the mother-child dyad interact, and that this impacts on a child’s health and wellbeing. This perception is based both on an infant’s characteristics and behaviour, and also on interpretations of these in relation to an ‘internal working model’ of attachment ^6^. MORS-SF measures the mother’s internal representation of their child in two dimensions: warmth-coldness (the ‘warmth’ sub-scale) and invasion-withdrawal (the ‘invasion’ sub-scale). It contains 14 statements derived from a previous 44-item scale (MORS), for each of which a mother is asked to rate agreement on a Likert-type scale from 0–5. It has previously been administered in English, Arabic, Dutch, Hungarian ^7, 8^, Polish, Russian and Chinese languages (translations available for download: https://www.morscales.org/mors-tools/). Previous work suggests that mothers who are stressed, anxious or depressed tend to score their infants more highly on the ‘invasiveness’ sub-scale of MORS-SF and this is likely to be problematic for infant wellbeing and development ^6, 7^. Mother’s and father’s MORS-SF scores for the same child are not highly correlated ^9^, supporting the assertion that the tool measures parental perception over child characteristics. MORS-SF has been validated against several measures of maternal reported infant temperament and maternal depression in both Britain and Hungary, including a crying/fussing diary, Mother and Baby Scales, the Infant Behaviour Questionnaire, Infant Characteristics Questionnaire, the General Health Questionnaire-12, Edinburgh Postnatal Depression Scale and the Cambridge Worry Scale ^6, 10, 11^.

In this paper we first describe our adaptation of MORS-SF for first-time use in India, and then present findings of how this tool relates to the Bayley Scales of Infant Development III (BSID-III) ^12^ - a comprehensive child developmental assessment tool - and HOME-IT ^13^, the most commonly used tool for measuring the quality of the home environment for child development, in a large population-based sample from the Early Life Stress (ELS) sub-study of the SPRING cluster randomised controlled trial (SPRING-ELS).

## Methods

### SPRING cluster randomised controlled trial: Early Life Stress sub-study (SPRING-ELS)

SPRING-ELS was a sub-study of the SPRING cluster randomised controlled trial in India. It explored the role of stress and adversity in early childhood and growth. SPRING-ELS methods are described in detail elsewhere ^12^, as are SPRING trial methods ( http://spring.lshtm.ac.uk, clinicaltrials.gov registration NCT02059863 11 ^th^ February 2014). Briefly, SPRING in India was an innovative, feasible, affordable and sustainable community-based intervention in Rewari district, Haryana, India aiming to improve early child growth and development through monthly home visiting by community-based workers. SPRING was evaluated by cluster randomised controlled trial. Clusters were defined by health sub-centre catchment areas. 12 clusters were allocated to the intervention arm, and 12 to control. Primary outcomes were the motor, cognitive and language scales of BSID-III ^14^ – and height-for-age, the best early childhood population-based proxy for human capital ^15^.

The study was conducted across 120 villages; total population was around 200,000. Rewari is predominantly rural and had health and demographic indicators around average for the state where the literacy rate was 76% (female literacy of 67%) ^16^. The sex ratio was 879 females per 1000 males ^16^ – amongst the lowest ratio in India. Infant mortality was 33/1000 live births ^17^ – slightly above the national average and similar to regional estimates. More than one third of under-five year old children were stunted (very low height-for-age) ^18^, this is amongst the highest stunting rates in the world.

The date of full implementation of SPRING was 18 June 2015. All children who were born after this date and lived in the study area were eligible for inclusion. Reasons for exclusion from the main SPRING trial were congenital anomaly (nine children) or mother not able to complete assessments (three children).

### Cultural adaptation of MORS-SF

The 14 MORS-SF statements are presented in Table 1. The tool was adapted for administration by local non-specialist female outcome assessors, resident in the study site for use when SPRING children were 12 months old.

**Table 1.** Fourteen Mother’s Object Relations Scales – Short Form (MORS-SF) items with summary of translation from English to Hindi.

Statement	Sub- scale	Original	Comments on translation
1	W	My baby smiles at me	Translation uncontroversial
2	I	My baby annoys me	We trialled two Hindi translations: *“mera bacha mujhe satata hai”* and *“mera bacha* *mujhe khija deta hai”* – the second was considered to be less accurate, meaning ‘imitating’ or ‘copying’. *“Satata hai”* implies a state of continuing annoyance without implication of action and was therefore chosen.
3	W	My baby likes doing things with me	The direct translation of ‘ *things’* (“ *cheez”)* was not appropriate to this sentence and needed to be explored in the sessions. Our suggestion of *“gatividhiya”* (‘ *activities’*) was equivalent but the first focus group participants were not sure on whether it would be understandable in the community. Subsequent sessions confirmed that “ *gatividhiya”* was both understood and acceptable.
4	W	My baby talks to me	Translation uncontroversial
5	I	My baby irritates me	Our original translation ( *“Mera baccha mujhe chida deta hai”*) was misunderstood by participants because the meaning of *“chida”* varies with context. Without further context the implication was widely understood as *‘teasing’*. The words *“jhunjhula”* and *“chidchidha”* were suggested by the two focus group discussion groups and tested in subsequent interviews. “ *Jhunjhula”* was selected because it is equivalent to *“irritate”* and was easily understood.
6	W	My baby likes me	Translation uncontroversial
7	I	My baby wants too much attention	This item presented a translation challenge as in English it is clear that *‘too much* *attentio*n’ implies negativity on the part of the respondent and is not equivalent to *‘a lot of attention’*. In Hindi we had to make this clear. *“Jarurat se jyada”* achieved this meaning.
8	W	My baby laughs	Translation uncontroversial
9	I	My baby gets moody	There was no directly equivalent word in Hindi for *‘moody’* - meaning a negative mood – so we trialed the English word *‘mood’* which we thought may be understood. Of note, in English it is implicit that this mood must be negative. In Hindi, this isn’t so clear and so we added *“kharaab”* (‘bad’) to clarify. Participants agreed and gave examples of child behaviours when a child has a negative mood.
10	I	My baby dominates me	We trialled three translation versions – “mera bacha muhe par haavi ho jaata hai”, “bacha mujhe kubad kar deta hai” and “mera bacha mujhe apni ungli par nachata hai”. The first was understood as *‘getting angry’* which wasn’t correct. The second was understood as *‘doing mischief’*, again not appropriate. The final is a local idiom which was understandable (direct translation: *‘my child makes me dance on their* *finger’*). There is a similar idiom in British-English: *‘my child has me wrapped around* *their little finger’*. The original tool author preferred using this idiom and agreed that it matches the underlying construct.
11	W	My baby likes to please me	Translation uncontroversial
12	I	My baby cries for no obvious reason	We tried various translations to account for the word ‘obvious’ which needed to account for simple things that young infants cry for – being hungry and so on. *“bina kisi baat ke”* ( *’no matter what’*) and *“bina kisi wajah”* ( *‘without any reason*’) were both acceptable translations but “ *bina kisi wajah”* was most equivalent to the original tool.
13	W	My baby is affectionate towards me	Our suggested translation *“mera bacha mere prati pyaar dikhata hai”*, was understood but the first focus group discussion suggested adding the word *“aur”* to emphasise that this affection must be towards the mother to count. This was understood in subsequent sessions.
14	I	My baby winds me up	This English idiom was the most difficult of the items to translate. The original tool author clarified that the underlying meaning is that the child repeatedly does things to raise the caregiver’s level of tension, so *‘winding up’* like for a clockwork motor. The emphasis is on the accumulative, and a suggestion that the child does it purposely. Our three potential translations were “Mera baccha intna pareshan”, “tang karta hai ki sehan” “bardast karna mushkil ho jaata hai”. We preferred *“tang”* to *“pareshan”* because it makes it clear that this word relates more to a child’s perceived mischievous behaviour over their actions in general, fitting with the tool author’s emphasis on the child’s purposive behaviour. We chose *“bardast”* over *“sehan karna”* to emphasise the implication of repeated actions leading to the ‘winding up’. The second focus group discussion understood the reformulated translation, with one participant giving an example: “when a mother is cooking food and the child keeps throwing things around even when told not to, repeatedly, it becomes intolerable for the mother”. In further sessions we found the translation to be working well.

Adaptation followed an adapted six step process ^19^:

***1) Translation into Hindi*** was carried out separately by DK (background in public health and mental health) and DV (background in public health and physical anthropology).

***2) Assessing technical equivalence*** was done by the adaptation team (SB, DV, DK, BK, RR, BRK, GD) in discussion with JO, the original MORS-SF author, and included a clinical psychologist (BK). The aim was to ensure that the translation matched the original meaning for each item. Where multiple translations achieved technical equivalence, all were field tested along with new suggested translations from team discussion.

***3) Field research:*** the aim was to assess ways in which translations were understood by respondents. This was done in two focus group discussions facilitated by DV and DK, first with SPRING non-specialist fieldworkers who were residents of the study area and were themselves mothers (approached because the study team felt they had the most insight into the topics being discussed), then with mothers from the population with children aged 2–5 years who were easier to access than mothers of infants but had recently raised children on this age (invited to participate by SPRING field supervisors). Both discussions were held in the SPRING site office. Facilitators explained that they wanted help selecting words that mothers of young children in Rewari would find it easy to understand. This process was: facilitator read out each statement and asked participants to repeat it using their own words. Where the participant’s description differed from the item meaning, the facilitator discussed this amongst the group and asked for improved wording.

***4) Finalisation of tool for pre-testing:*** field results (summarised in Table 1) were reviewed by the adaptation team with contribution from original tool author JO when required, and a revised tool prepared for pretesting.

Back translation was performed by an independent bilingual public health specialist; results were excellent and no changes were needed.

***5) Pretesting*** was done with eight mothers of children aged 2–5 years (convenience sample) individually in their home, by DV accompanied by a note-taker. Participants understood the translations and concepts and were able to give examples of each item. Field notes document that mothers had expressive faces which matched their answers – for example, in response to the statement “my child smiles at me”, a mother smiled back at the research associate. A small clarification was needed for item 3 - in response to the statement “my child likes doing things with me” two mothers asked “what type of things?”. We therefore edited the training manual that if asked, assessors should indicate that this applies ‘to all things’.

**Figure 1.**
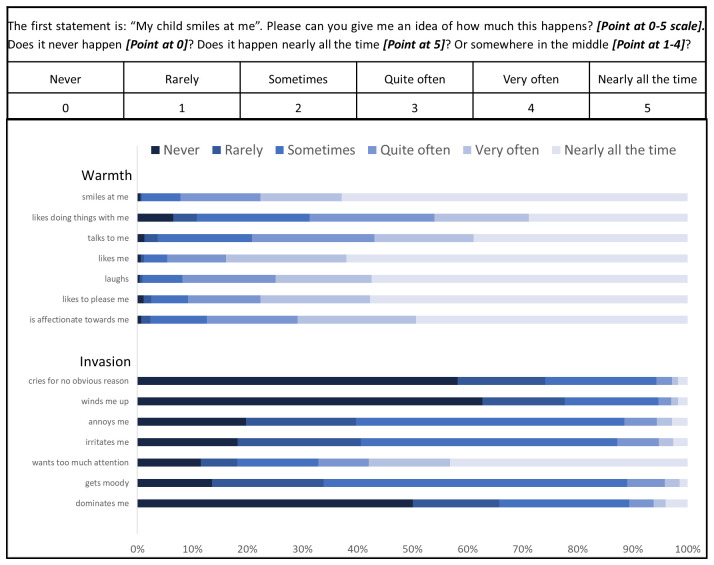
Panel A: Mother’s Object Relations Scales – Short Form (MORS-SF) instructions to be read by assessor printed on the form. Actions outlined in bold. Mothers were asked to point at the box which most clearly represented their response to each item. Panel B: MORS-SF: percentage of responses to each of 14 items grouped within the warmth and invasion sub-scales.

Pretesting also addressed administration of the tool. We presented the Likert-type scale presented in Figure 1 to mothers and read a statement asking them to point at the number they most agreed with. Mothers understood the system and it was quick to administer.

***6) Training & pilot-testing***: 12 assessors were trained by DV, DK and SB using didactic methods and role-play. Pilot-testing was done alongside other tools being administered at the 12-month assessment. 12 assessors worked in pairs and visited mothers at home, which also allowed them to become familiar with travelling around the study site, and to appreciate some of the challenges associated with assessing mother-child dyads in the home. Each assessor conducted two assessments with mothers of children aged 11–13 months old. Their partner scored the same assessment simultaneously, giving a total of four assessments per pair. Inter-rater reliability was greater than 95% for all six assessor-pairs. All assessors were observed performing a minimum of one assessment by a member of the adaptation team.

### Data collection

Children and their mothers were assessed at 12 and 18 months of age as described below. All assessments were done in the child’s home. Data were collected on paper forms which were double-entered and verified using a computer program written in C Sharp with an SQL Server 2008 database. EpiData is an open-source alternative which could be used for the same purpose.

### Explanatory variables

We visited families when enrolled children were 12 months of age to administer a range of SPRING and SPRING-ELS assessments including MORS-SF. There were three MORS-SF explanatory variables which are part of the original MORS-SF scale. What MORS-SF describes as a ‘warmth sub-scale’ score was calculated according to the manual by summing the scores of the seven warmth items giving a score of 0–35. Similarly, what it describes as ‘invasion’ scores were calculated by summing the scores of the seven invasion items. In addition, MORS-SF defined ‘concern’ levels were calculated using a combination of these two sub-scale scores shown in Table 2 and described in the footnote (these levels were described by Milford *et al.* ^20^ and not altered for this study).

**Table 2.** MORS-SF concern categories: association with child developmental outcomes.

MORS concern category	Warmth score	Invasion score	n	%	Predicted mean (95% CI) BSID-III scores			Predicted mean (95% CI) HOME- IT scores
					Motor	Cognitive	Language	
None	26-30	0-6	217	17.1	96.3 (94.6, 98.0)	93.5 (91.5, 95.4)	92.7 (89.7, 95.6)	32.6 (31.6, 33.6)
Low	23-25 23-35	0-6 7-11	415	32.6	95.1 (91.8, 98.4)	93.1 (89.4, 96.7)	91.0 (85.8, 96.2)	32.2 (30.6, 33.8)
Moderate	23-35 0-22	12-35 0-11	582	45.7	93.3 (90.1, 96.6)	91.6 (88.0, 95.2)	89.6 (84.5, 94.7)	31.8 (30.2, 33.4)
High	0-22	12-35	59	4.6	91.7 (87.2, 96.1)	89.7 (84.7, 94.6)	83.8 (76.9, 90.7)	29.9 (27.8, 32.0)
			p-trend		<0.001	0.025	<0.001	<0.001
			Overall mean (SD)		94.5 (9.7)	92.6 (10.6)	90.7 (14.2)	31.6 (4.1)

Note that these categories were defined earlier and have not been altered for this work. Categories are: None (warmth 26-30 points and invasion 0-6); Low (warmth 23-25 points and invasion 0-6; or warmth 23-35 points and invasion 7-11); Moderate (warmth 23-35 points and invasion 12-35; or warmth 0-22 points and invasion 0-11); High (warmth 0-22 points and invasion 12-35 points). MORS-SF: Mother’s Object Relations Scales – Short Form; BSID-III: Bayley Scales of Infant Development III; CI, confidence interval; SD, standard deviation.

### Outcome variables

Assessors did the 45-item HOME-IT assessment at the 12-month visit to assess the degree to which the home environment was supportive of child development. HOME-IT uses a mixture of questions to the mother and assessor observations and is done over an hour. Each item is scored 0 or 1. Some items can only be scored positively if observed, e.g. “Mother spontaneously praises child at least twice”. Others are asked directly e.g. - “Does child’s father or father figure help with looking after [child’s name]?”.

Assessors did child development assessments at 18 months of age using the motor, cognitive and language scales of the BSID-III in the home ^14^. They did two BSID-III assessments per day in pairs. Each assessment took 2–3 hours to complete. Each BSID-III scale consists of a series of progressively more difficult activities which children are asked to do whilst interacting with an assessor. Each item was scored 1 if the activity was demonstrated, otherwise it was scored 0. Assessment on each scale started at the item marked ‘K’ (start point for 16.5 – 19.5 month old children). We did comprehensive cultural adaptation and inter-rater reliability (IRR) checks, finding mean agreement between assessors of greater than 97%.

The same adaptation process described for MORS-SF was used for HOME-IT. For BSID III we did comprehensive cultural adaptation and inter-rater reliability checks finding mean agreement between assessors of greater than 97%.

### Statistical methods

Sample size was determined by the requirements of the main SPRING trial, which aimed with 90% power to detect a minimum effect size of 0.38 given an assumed intra-cluster correlation of 0.05 and significance level of 0.05. We therefore assessed all children being assessed for SPRING which was at least the first 50 children born in each of the 24 clusters from 18 June 2015 (minimum total sample size 1200).

We initially used mixed effects linear regression to assess the association between the three explanatory variables and the four outcome variables. All models were adjusted for the clustered design of the SPRING trial by including cluster as a random effect, and for trial arm as a fixed effect to account for any impact of the intervention. We next predicted values for the outcome variables for interacting values of the explanatory variables ‘warmth’ and ‘invasion’ using these models and present these results both in tabular and also graphical form.

We used Stata 15 for all statistical analyses (StataCorp LLC: College Station, TX, USA).

### Ethics

Approval was obtained from the London School of Hygiene & Tropical Medicine research ethics committee (23 June 2011, approval number 5983; 19 May 2015, approval number 9886) and the Sangath Institutional Review board (IRB) (19 February 2014; 27 May 2015). Approval was also granted by the Indian Council of Medical Research’s Health Ministry Screening Committee (HMSC) (24 November 2014; 6 October 2015). Informed written consent was obtained from mothers at enrolment into the trial surveillance system and again before a child’s first birthday for detailed assessments.

## Results

### SPRING-ELS sub-sample

1726 liveborn babies were recruited into SPRING-ELS. Of these, 1273 (73.8%) completed 12- and 18-month assessments ^21^. Children who missed either were consider lost to follow-up. The flowchart describing this is presented elsewhere ^12^ together with a table showing no evidence of selection bias comparing those included versus lost to follow-up with respect to socioeconomic status, caste, sex, multiple pregnancy, being delivered in a facility, and mother’s education and age. Reasons for loss to follow-up include not being available for assessment at 12 months (12.0%) or 18 months (0.8%), consent refusal (6.1%), having moved away (4.8%), and death of mother or child (2.6%).

### MORS-SF Responses

Figure 1 shows considerable variation in responses to MORS-SF statements. In the warmth sub-scale the majority of mothers responded ‘nearly all the time’ to statements: “My baby… ‘smiles at me’ (826 mothers; 62.9%), ‘likes me’ (814; 62%), ‘laughs’ (754; 57.4%), and ‘likes to please me’ (758; 57.7%)”. These very positive ratings were less frequent for the statements: “My baby… ‘likes doing things with me’ (379; 28.9%), ‘talks to me’ (511; 38.9%) and ‘is affectionate towards me’ (648 (49.4%)”. In the invasion sub-scale, most mothers responded ‘never’ to the statements: “my child winds me up” (823; 62.7%) and “my child dominates me” (657; 50%); however, a considerable proportion indicated in response to the statements “my baby annoys me” and “my baby irritates me” that this happens rarely, sometimes or quite often (980; 74.6%, and 1005; 76.5% respectively). Many mothers responded to the statement “my baby wants too much attention” with ‘nearly all of the time’ (567; 43.2%).

**Figure 2.**
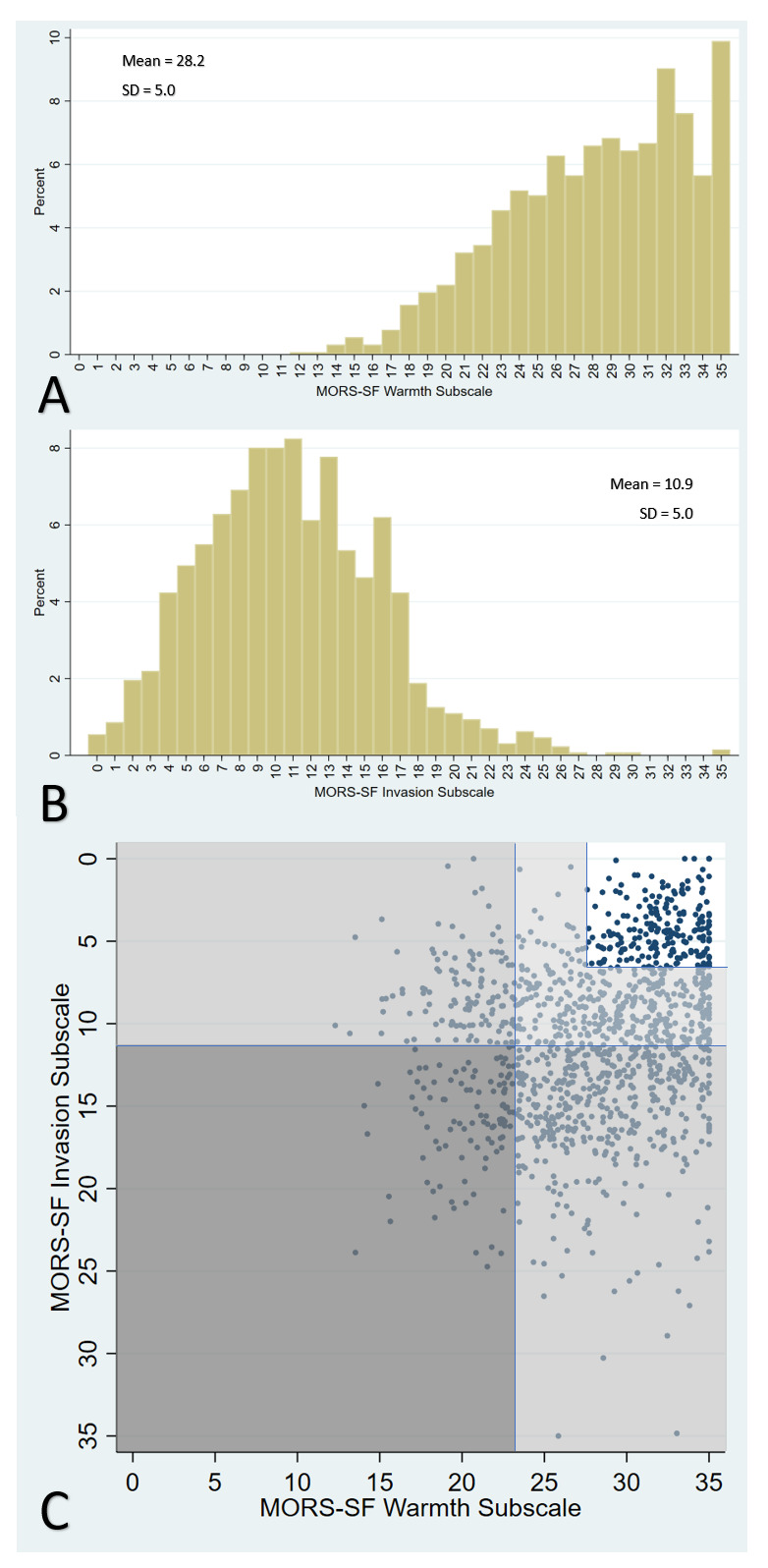
Mother’s Object Relations Scales – Short Form (MORS-SF): histograms showing distribution of scores in the Warmth (Panel A) and Invasion (Panel B) sub-scales and scatterplot of relationship between these scores, including categorisation of concern levels shaded in dark grey (high), medium grey (moderate) light grey (low) and white (no concern) (Panel C).

The mean warmth score was 28.2 and invasion score was 10.9 (possible range 0–35). Figure 2 shows histograms for warmth and invasion scores, and illustrates the relationship between these scores. 17.1% of dyads were categorised as ‘no concern’ in the relationship, 32.6% as low-concern, 45.7% as moderate-concern and 4.6% as high concern; the majority of moderate-concern categorisation was due to high invasion scores (508 children) rather than low warmth (74 children) ( Figure 2C).

### Association of MORS-SF with child development indicators

Mean HOME-IT score was 31.6. Mean BSID-III scores were 94.5, 92.6 and 90.7 for the motor, cognitive and language domains.

**Figure 3.**
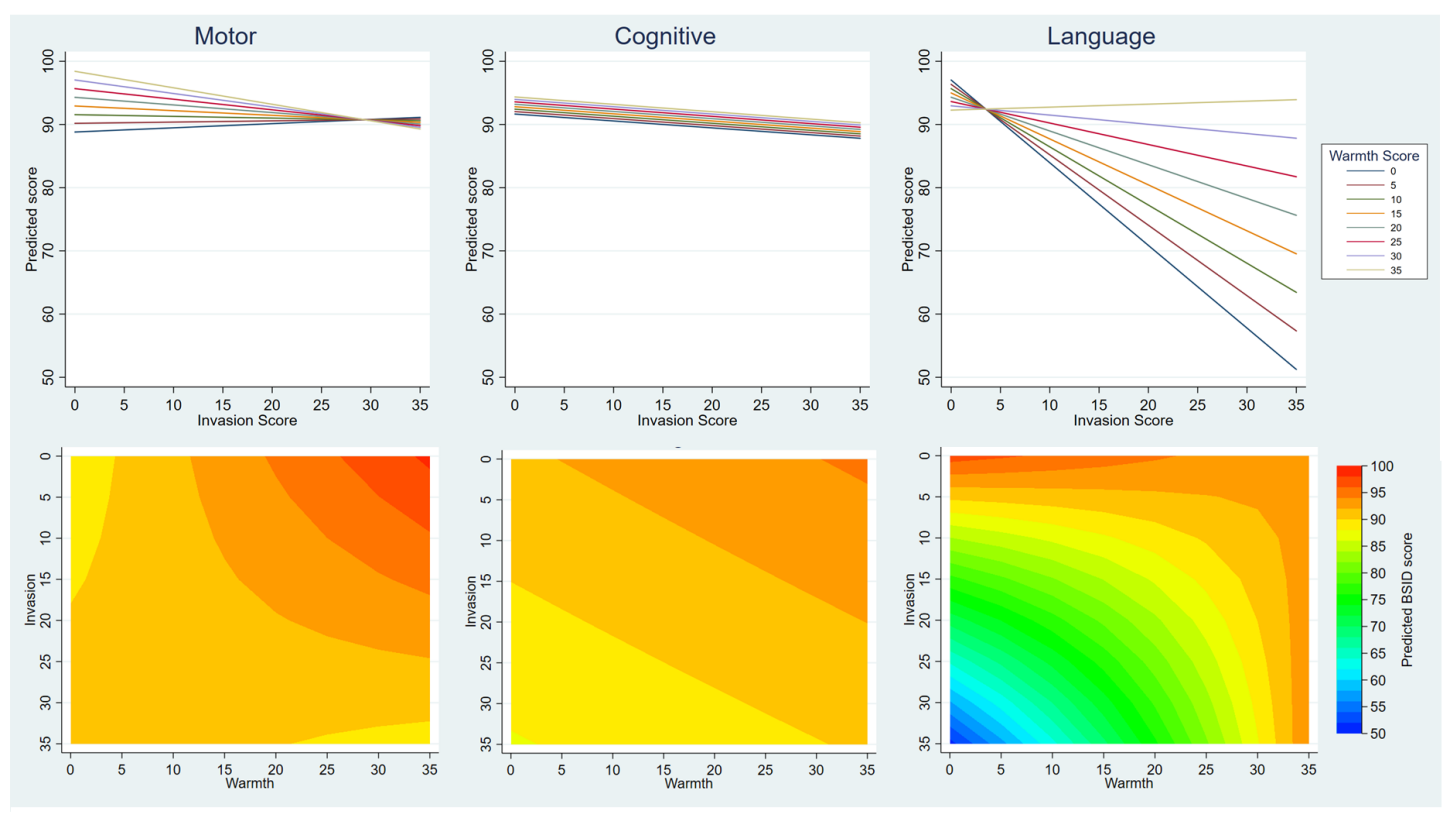
Illustration of modelling showing association between Mother’s Object Relations Scales – Short Form (MORS-SF) and Bayley Scales of Infant Development III (BSID-III). The upper line graphs show the interaction of the effects of invasion and warmth on child development – the y axis shows the model predicted scores in the motor cognitive and language domains, the x-axis shows how this varies by invasion, and the coloured lines show how this varies by warmth. The lower contour plots show how predicted BSID scores (contoured colours) are affected by the interaction of warmth and invasion scores (x and y axes). The more curved the contours, the greater the interaction between these two variables.

For the motor and language BSID-III scales and for HOME-IT there were strong and positive associations with the warmth sub-scale (for each point increase in warmth, an increase of 0.22 (95% CI 0.10, 0.33) points on the motor-scale, 0.33 (95% CI 0.17, 0.48) for language, 0.08 (95% CI 0.04, 0.13) for HOME-IT); and similar magnitude strong and negative associations with the invasion sub-scale. Similar associations were seen with the BSID-III cognitive scale but with smaller point estimates and wider confidence intervals ( Table 3A). Table 3B shows the results of models including both warmth and invasion; these also included an interaction term to allow for the possibility that these two explanatory variables did not act independently. The models for BSID-III motor and language scales show strong evidence of interaction which is illustrated in Figure 3 in two ways. In the top half of the figure, each of the eight lines on the graph represent a warmth sub-scale score in five point increments from 0 to 35. The invasion sub-scale score is plotted on the x-axis. The predicted BSID-III score for any given combination of these scores can then be read from the y-axis, showing that compared with the motor and cognitive scores, there is a much larger difference in the language score as warmth scores change. These graphs also show that higher invasion scores are associated with lower BSID-III scores overall, but that this relationship was attenuated by high warmth, particularly in motor and language BSID-III scales. The contour plots in the bottom half of Figure 3 illustrate these same relationships in a different way, giving the predicted BSID-III score in a colour for any given level of invasion (read on the y axis) and warmth (read on the x axis). The strong interaction shows on these graphs as curved lines in the language and motor scales, whereas for cognitive the boundary lines are parallel which is indicative of no interaction.

**Table 3.** MORS-SF: Panel A - Association between each of warmth & invasion sub-scale and child developmental outcomes (adjusted for clustering and SPRING trial allocation).

A	Overall mean (SD)	Warmth models			Invasion models		
		Mean at 0 (95% CI) *	Change for increase (95% CI)	p	Mean at 0 (95% CI) *	Change for increase (95% CI)	p
Motor	94.5 (9.7)	88.2 (84.9, 91.5)	0.22 (0.10, 0.33)	<0.001	96.9 (95.2, 98.5)	-0.23 (-0.34, -0.13)	<0.001
Cognitive	92.6 (10.6)	89.5 (85.8, 93.1)	0.10 (-0.02, 0.22)	0.099	93.7 (91.8, 95.6)	-0.13 (-0.25, -0.02)	0.026
Language	90.7 (14.2)	81.1 (76.1, 86.1)	0.33 (0.17, 0.48)	<0.001	93.2 (90.3, 96.1)	-0.27 (-0.42, -0.12)	0.001
HOME-IT	31.6 (4.1)	29.7 (28.2, 31.2)	0.08 (0.04, 0.13)	<0.001	32.8 (31.9, 33.8)	-0.08 (-0.12, -0.04)	<0.001
B	Warmth & Invasion models						
	Mean at Warmth=0 & Invasion=0 (95% CI)	Warmth		Invasion		Interaction term	p model
		Slope *	P	Slope *	p		
Motor	88.7 (81.1, 96.3)	0.27 (0.02, 0.53)	0.035	0.07 (-0.55, 0.68)	0.832	-0.009 (-0.03, 0.01)	<0.001
Cognitive	91.4 (83.0, 99.8)	0.08 (-0.20, 0.36)	0.589	-0.11 (-0.78, 0.57)	0.750	-0.0002 (-0.02, 0.02)	0.150
Language	96.7 (85.7, 107.7)	-0.14 (-0.50, 0.23)	0.466	-1.31 (-2.19, -0.42)	0.004	0.039 (0.008, 0.069)	<0.001
HOME-IT	32.0 (28.9, 35.2)	0.02 (-0.08, 0.13)	0.645	-0.18 (-0.43, 0.07)	0.156	0.004 (-0.005, 0.013)	<0.001

*Mean outcome score when explanatory variable (warmth or invasion) is equal to zero. Panel B - Association between warmth and invasion sub-scales in one model and child developmental outcomes. *Slope when other explanatory variable (warmth and invasion) is equal to zero. MORS-SF: Mother’s Object Relations Scales – Short Form; CI: confidence interval; SD: standard deviation.

**Figure 4.**
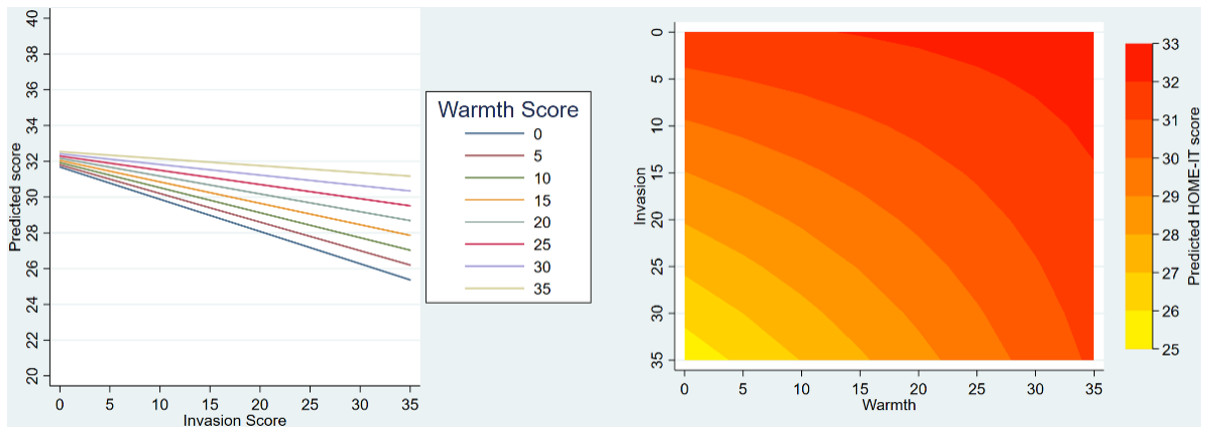
HOME-IT illustration of final models including interaction term. The left-hand graph shows the interaction of the effects of invasion and warmth on HOME-IT – the y axis shows the model predicted score, the x-axis shows how this varies by invasion, and the coloured lines show how this varies by warmth. The right-hand side (contour plot) shows how predicted HOME-IT score (contoured colours) is affected by the interaction of warmth and invasion scores (x and y axes). The curved lines suggest interaction between these two scores.

Similar illustrations are presented in Figure 4 for HOME-IT; on the left side, lines representing warmth scores diverge as the invasion score increases, and on the right side the contour plot shows that the boundary lines between predicted outcome scores are curved. Both of these illustrate the strong evidence that warmth and invasion scores interact with respect to HOME-IT scores.

### Association of MORS-SF level of concern with child development indicators

**Figure 5.**
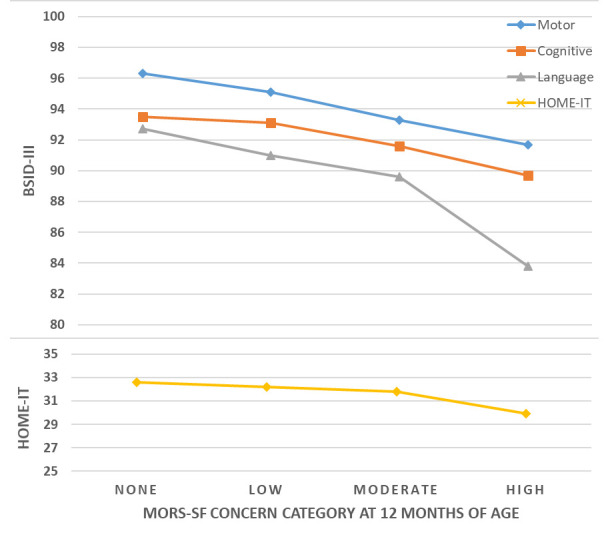
Association between Mother’s Object Relations Scales – Short Form (MORS-SF) concern category at 12 months of age and Bayley Scales of Infant Development III (BSID-III) developmental domain scores at 18 months of age.

There were consistent and negative associations between MORS-SF concern levels and BSID-III and HOME-IT scores. Between MORS-SF high and no concern levels this associated decrease in HOME-IT score was 2.5 points (approximately 0.5SD), nearly 9 points in the BSID-III language domain (0.6SD), nearly 4 points in the cognitive domain (0.35SD), and 4.6 points in the motor domain (0.47SD). These relationships are outlined in Table 3 and are illustrated in Figure 5, which shows decreasing developmental scores as MORS-SF concern level increases from no-concern to high-concern.

## Discussion

We adapted the MORS-SF for use with mothers in Haryana, India and found that scores at 12 months of age were associated with lower concurrent HOME-IT scores indicating a lower quality of the home environment, and with lower infant development scores in the motor, cognitive and language domains of the BSID-III scale measured at 18 months of age. This was the first time that MORS-SF has been examined with respect to these child developmental assessment tools and our study provides new insights into a tool for measuring the mother-infant relationship in community-based settings in LMICs. This is particularly important as the child survival agenda expands to incorporate nurturing care and early childhood development in these settings, with an emphasis on improved carer-child interactions and increasing carer responsiveness, both of which are closely related to a mother’s working model of her infant as explored in MORS-SF.

The finding that warmth scores were reasonably high and contribute relatively less to MORS concern levels than the invasion scores is noteworthy, suggesting that whilst mothers may feel warm and affectionate towards their infants, they may nevertheless feel that their infants overly impinge on their own being (e.g. by endorsing statements such as ‘my baby annoys me’ and ‘my baby irritates me’). Our group’s previous work in this area of India found that mothers are busy and stretched for time ^22^ and it is possible that this accounts for some of this feeling of invasion when interaction with young babies is one amongst a range of activities competing for a mother’s time. This possibility is backed up by the >40% of mothers responding with “nearly all the time” to the statement “My baby wants too much attention”. Both the warmth and invasion scores are similar to those presented in the original MORS-SF British cohort when infants were 2–6 months old (warmth mean (SD): 29 (3.7); invasion mean (SD): 11.3 (4.3)). Warmth scores are similar to a Hungarian cohort of 97 mothers when infants were 6 months of age (mean (SD): 28.4 (3.8)), however invasion scores are a little higher (mean (SD): 7.8 (3.9)). This general consistency across the three diverse settings of the United Kingdom, Hungary and India is striking and lends support to the contention that (with rigorous adaptation) this is a tool that could be of use in multi-country assessments for child wellbeing.

Possible reasons for the MORS-SF and HOME-IT associations include that mothers who have high warmth or low invasion scores on MORS-SF may score higher in the mother-child interaction section of HOME-IT. It is also possible that there are material changes in home environments, for example of play materials connected to the mother’s internal representation of her child. The overall finding is useful because HOME-IT is difficult to use at-scale.

The finding that the relationship of BSID scores was similar with each of the warmth and invasion sub-scales in initial models suggests that these individual sub-scales are equally important for predicting development. The final model including the interaction term shows that for language, at any given level of invasion score, a mother’s high warmth score of 35 is associated with the highest language score (and similarly for other very high warmth scores). For the motor scale, the relationship is the opposite; the model predicts that if warmth is low then invasion score has minimal influence on motor scores. This suggests that combining the two sub-scales is crucial to use of MORS-SF.

This is the first time that the MORS-SF has been examined with respect to child development. Our results fit within the larger body of literature supporting the role of parental affect, the mother-child relationship and attachment status in affecting child developmental outcomes ^23– 25^. Strengths include that this is the largest sample to have been assessed using MORS-SF. We adjusted for possible clustering in the data, and for allocation to trial group because of possible confounding.

MORS-SF could be a valuable addition to early childhood development research and programmes in LMICs because of: a) its focus on assessing the mother’s perceptions of her child in the warmth and invasion domains, which may be useful to encouraging improved responsiveness; and b) the feasibility of administration at scale.

Cultural adaptation required a team with in-depth understanding of at least two cultures, multiple languages and of the tool. Careful fieldwork was needed to understand how to best adapt specific meanings to a new context. We allocated time and resources to produce a tool that made sense to participants and gave reliable results. Whilst no consensus exists on the ideal format of cross-cultural validation studies ^26^, the strengths of our approach are well described and include: 1) translation involving a clinical psychologist with considerable understanding and experience of test development principles ^27^; 2) reconciliation of two independent translations ^28^; 3) multidisciplinary and multilingual review by multiple persons ^29^; 4) avoidance of over-reliance on back-translation ^26, 30^; 5) focus on the meaning of individual words and items in focus groups and cognitive interviews ^27, 29, 31^; 6) involvement of the original tool author ^32^; and 7) pre-testing by a research associate with understanding of the tool, the adaptation process and both languages ^26^. The possibility of translations not being exact matches means that care should be taken, and further work done, if translated tools are to be used in multicountry (or multi-language in a single country) studies. We found the cognitive interviews and pre-testing to be of crucial importance and highly commend this approach to those conducting further work on the tool in varied languages.

Design limitations of this study include that we administered MORS-SF with mothers only, but we recognise that much care is provided by the wider family. Future work in such a context could assess other family members’ responses to MORS-SF to more deeply understand the contribution of each family member. Whilst our data provides good cross-sectional evidence, future work is needed to further determine causality including the possibility of unmeasured confounding, for example by socioeconomic status, which was not accounted for in these analyses. Additional future work will include examination of convergent validity with respect to other relational risk-factors, and further formal psychometric validation.fv

We found that MORS-SF - a standardised measure of a mother’s perception of her infant - could be culturally adapted for use in rural India, and that the results of this adapted tool were associated with indicators of early childhood development. MORS-SF is short, understandable and has shown potential for incorporation into studies examining responsive caregiving and early childhood, particularly for the majority of the world’s children who live in LMICs.

## Data availability

### Underlying data

LSHTM Data Compass: SPRING Early Life Stress Sub-study: Additional Resources, https://doi.org/10.17037/DATA.00002095 ^21^.

This project contains the following underlying data:

- SPRING-ELS_dataset.csv (The dataset contains information collected from 1,273 children who were enrolled in the substudy. The dataset contains an anonymised participant ID number, cluster codes, trial arm allocation, and the scores for each child.)
- SPRING-ELS_dataset_codebook.html (Codebook for the SPRING-ELS dataset)

### Extended data

LSHTM Data Compass: SPRING Early Life Stress Sub-study: Additional Resources. https://doi.org/10.17037/DATA.00002095 ^21^.

This project contains the following extended data:

- ELSQUS_Hindi.pdf (Early life adversity questionnaire in Hindi; PDF format; administered at 12m assessment; includes MORS-SF)
- ELSQUS_English.pdf (Early life adversity questionnaire English translation; PDF format)

Data are available under the terms of the Creative Commons Attribution 4.0 International license (CC-BY 4.0).
