# Supplementary material for: Using the Mothers Object Relations Scale for early childhood development research in rural India: Findings from the Early Life Stress Sub-study of the SPRING Cluster Randomised Controlled Trial (SPRING-ELS)
Source: Wellcome Open Res. 2022 Jan 31;6:54. Originally published 2021 Mar 10. [Version 2] doi: 10.12688/wellcomeopenres.16591.2 (PMC8847700; doi:10.12688/wellcomeopenres.16591.2)
Supplement: Peer review history

# Peer review: Reviewer response for version 2

**DOI:** 10.21956/wellcomeopenres.19473.r48361

**Published:** 2022-02-15

**Authors:** Ming Wai Wan

**Affiliations:** 1 University of Manchester, Manchester, UK

My only minor point in response to the authors' response/amendments is this:

  

- "In the invasion sub-scale, most mothers responded ‘never’ to the statements: “my child winds me up” (823; 62.7%) and “my child dominates me” (657; 50%)"
  Technically, regarding the latter, 50% is not 'most mothers'! I suggest minor modification of wording

Is the work clearly and accurately presented and does it cite the current literature?

Yes

If applicable, is the statistical analysis and its interpretation appropriate?

Yes

Are all the source data underlying the results available to ensure full reproducibility?

Yes

Is the study design appropriate and is the work technically sound?

Yes

Are the conclusions drawn adequately supported by the results?

Partly

Are sufficient details of methods and analysis provided to allow replication by others?

Yes

Reviewer Expertise:

NA

I confirm that I have read this submission and believe that I have an appropriate level of expertise to confirm that it is of an acceptable scientific standard.

# Peer review: Reviewer response for version 2

**DOI:** 10.21956/wellcomeopenres.19473.r48362

**Published:** 2022-02-08

**Authors:** Anna Kamza

**Affiliations:** 1 Institute of Psychology, SWPS University of Social Sciences and Humanities, Warsaw, Poland

Thank you very much for your responses. I have no further comments to make.

Is the work clearly and accurately presented and does it cite the current literature?

Partly

If applicable, is the statistical analysis and its interpretation appropriate?

Yes

Are all the source data underlying the results available to ensure full reproducibility?

Yes

Is the study design appropriate and is the work technically sound?

Yes

Are the conclusions drawn adequately supported by the results?

Yes

Are sufficient details of methods and analysis provided to allow replication by others?

Yes

Reviewer Expertise:

Cognitive psychology, Individual differences, Developmental Psychology, Attachment, Mentalization, Executive Function, Parental Reflective Functioning, Parental Burnout.

I confirm that I have read this submission and believe that I have an appropriate level of expertise to confirm that it is of an acceptable scientific standard.

# Peer review: Reviewer response for version 1

**DOI:** 10.21956/wellcomeopenres.18285.r46790

**Published:** 2021-11-12

**Authors:** Anna Kamza

**Affiliations:** 1 Institute of Psychology, SWPS University of Social Sciences and Humanities, Warsaw, Poland

Thank you for the opportunity to read the manuscript "Using the Mothers Object Relations Scale for early childhood development research in rural India (...)". In my opinion, the Authors did a really good job.

 However, I have some brief suggestions that might help to enrich the paper.

 Please, provide a brief and clear theoretical background for the Mothers Object Relations Scale. Although the Authors mention that "It has a basis in attachment theory and attempts to measure an element of responsive caregiving, both of which are crucial for optimal child development." (p. 3), I think a little more detailed description would help the readers who are not familiar with the attachment theory.

 Please, delete the horizontal lines in Figure 5.

 Some detalied  references to the MORS adaptations in other languages (i.e., English, Hungarian, Polish and Chinese) should be provided.

 Are there any limitations of the MORS usage (e.g., in terms of SES background etc.)? I think it is worth to take up this topic in the Discussion section.

 Finally, a proofreading is needed throughout the paper.

Is the work clearly and accurately presented and does it cite the current literature?

Partly

If applicable, is the statistical analysis and its interpretation appropriate?

Yes

Are all the source data underlying the results available to ensure full reproducibility?

Yes

Is the study design appropriate and is the work technically sound?

Yes

Are the conclusions drawn adequately supported by the results?

Yes

Are sufficient details of methods and analysis provided to allow replication by others?

Yes

Reviewer Expertise:

Cognitive psychology, Individual differences, Developmental Psychology, Attachment, mentalization, Executive Function.

I confirm that I have read this submission and believe that I have an appropriate level of expertise to confirm that it is of an acceptable scientific standard, however I have significant reservations, as outlined above.

# Response

**Published:** 2022-01-11

**Authors:** Sunil Bhopal

Thank you for the opportunity to read the manuscript "Using the Mothers Object Relations Scale for early childhood development research in rural India (...)". In my opinion, the Authors did a really good job. However, I have some brief suggestions that might help to enrich the paper.

**Thank you for these comments and for taking the time to read our paper. We really appreciate this.**

 Please, provide a brief and clear theoretical background for the Mothers Object Relations Scale. Although the Authors mention that "It has a basis in attachment theory and attempts to measure an element of responsive caregiving, both of which are crucial for optimal child development." (p. 3), I think a little more detailed description would help the readers who are not familiar with the attachment theory.

**We have added to the background to MORS with an additional sentence. This section now reads:**

**“The Mother’s Object Relations Scales – Short Form (MORS-SF) was developed in the 1990s for assessing areas of difficulty in the early mother-infant relationship** **^5^** **. It has a basis in attachment theory and attempts to measure an element of responsive-caregiving, both of which are crucial for optimal child development. The premise is that a mother’s perception of her infant influences the ways in which the mother-child dyad interact, and that this impacts on a child’s health and wellbeing. This perception is based both on an infant’s characteristics and behaviour, and also on interpretations of these in relation to an ‘internal working model’ of attachment.”**

**Along with an additional reference to further descriptions in a detailed paper on the subject, for those who wish to learn more about attachment and the underlying developmental psychology.**

 Please, delete the horizontal lines in Figure 5.

**We trialled doing this but the effect was to make the graph difficult to read across from the y-axis. For this reason we would prefer to leave as it is pending editorial advice.**

 Some detalied  references to the MORS adaptations in other languages (i.e., English, Hungarian, Polish and Chinese) should be provided.

**These tools are now available to download in Arabic, Chinese, Dutch, Hindi, Hungarian, Polish, Russian. We have added a reference to the website for download -** **https://www.morscales.org/mors-tools/** - **and added references to the literature where the Hungarian version was used (introduction; paragraph 3)**

 Are there any limitations of the MORS usage (e.g., in terms of SES background etc.)? I think it is worth to take up this topic in the Discussion section.

**We have now added this point to the limitations section and caveated further throughout the discussion section**

 Finally, a proofreading is needed throughout the paper.

**Thank you. We have proofread the paper again and made corrections in tracked-changes throughout.**

# Peer review: Reviewer response for version 1

**DOI:** 10.21956/wellcomeopenres.18285.r44740

**Published:** 2021-08-02

**Authors:** Ming Wai Wan

**Affiliations:** 1 University of Manchester, Manchester, UK

This paper examines a Hindi version of a brief self-report measure of parental perceptions of their relationship (warmth, invasiveness) toward their 12-month-old infant in rural India. This version of the Mother-Object Relations Scale (MORS-SF) was utilised in a published clinical trial on preventing the impacts of early life stress (Bhopal *et al.*, 2019), and this secondary paper describes how well the measure compares with 18-month developmental (e.g. cognitive, language, home environment quality) outcomes, as well as describes some of the psychometric characteristics of this translated version. The paper has potential high importance given what is known about how early parent-child relationships impact on various developmental outcomes, given how early stress that is very common in LMIC countries impacts on such relationships and given the ease of implementing this kind of measure in a low resource environment. The sample size obtained is very large for a study of this kind.

 The introduction sets out the rationale very well and is accurate and impressively concise in presentation. While the MORS-SF has been available and in use for a while, the empirical literature seems not have caught up with that. For example, while the cited studies are of some relevance, the papers are: (1) a conference presentation – can details be provided for accessing the presentation poster or slides? (2) a Warwickshire (UK) study – while detailed, it applies to a different age group (2-4 years) and the number scales are modified for analysis; (3) a Hungarian study focused on postpartum depression and anxiety at 8-12 months postpartum; and (4) the aforementioned trial in which the MORS-SF is utilised as a screener with the current sample; those with moderate to high levels of concern were considered one of four relational risk factors (so no analysis details are provided specific to the MORS-SF). Therefore, while the MORS-SF is somewhat established and well-known, the psychometric data for the English language version for the relevant child age group is not as available or accessible as perhaps suggested. This clearly limits any ability to compare the findings here with other similar samples from different cultural contexts, including the UK where the measure originates. Perhaps further citations could be added to strengthen where possible, or there could be some acknowledgement in the limitations or as a general discussion point. A specific citation could be added to the assertion that mothers’ and fathers’ MORS-SF scores for the same child are not highly correlated.

 The study design is appropriate, though it tries to be both a methodology and primary research paper. I applaud that the categories of concern as derived from the MORS-SF are provided here (Table 3) where they were not in Bhopal et al. (2019). The footnote of Table 3 states that the categories were defined prior to this study. It would be helpful to have clarification on how the categories of concern were defined and, if possible, a citation could be provided to support that. While there is certainly merit in studying the MORS-SF subscales in relation to these 18-month outcomes, it would also have seemed particularly appropriate (given the pre-validation stage of this translated version) to examine the MORS-SF in relation to the other 3 relational risk factors (mentioned in Bhopal et al., 2019) to give some indication of convergent validity. It would seem appropriate to discuss this somewhere in the discussion – perhaps as a limitation or as an important consideration for future research.

 The methods and analysis are detailed very well and are replicable. It would help the reader by offering Ns and % in the paragraph explaining MORS-SF responses (i.e. highlights of Figure 1). The figures are interesting and generally well explained in the text. The detail of the translation process is very good yet concise, and the Hindi version of the measure is available for others to use. All source data is available.

 The conclusions drawn are mostly adequately supported by the results, though this section could be strengthened by being a little more balanced in presentation and with the findings interpreted more critically. With regards to the former, there is a strong focus on the strengths of the study and the measure. I appreciate that one aim of the paper is surely to promote the viability of this brief measure, especially for use in LMIC contexts. Still, critical interpretation of the findings could be enriched and the limitations more thoroughly considered (ideas already mentioned above). One could unpack the different possible reasons for MORS-SF links with the later outcomes – for example, both variables could be linked to a third variable, such as socioeconomic deprivation. Were any variables controlled for? When considering translation, I appreciate that efforts went into ensuring that the meaning was maintained in the Hindi version. Given that this is a new translated version, I do wonder if there were still any concerns about some meaning being lost in translation. For example, many mothers agreed strongly that ‘my baby wants too much attention’ – it is possible that the connotations of this sentence could really differ across cultural contexts, with some interpretations being more negative towards the child than others might. Some reflection on translation issues could serve well for future usage and research, as well as translations into other languages, and for use in multicultural groups in clinical contexts. Considerations for the breadth of the audience will only serve to enhance what this paper already has to offer.

 Finally, thank you for the opportunity to review this interesting paper focused on an under-researched population with respect to parent-infant relationships and child outcomes.

Is the work clearly and accurately presented and does it cite the current literature?

Yes

If applicable, is the statistical analysis and its interpretation appropriate?

Yes

Are all the source data underlying the results available to ensure full reproducibility?

Yes

Is the study design appropriate and is the work technically sound?

Yes

Are the conclusions drawn adequately supported by the results?

Partly

Are sufficient details of methods and analysis provided to allow replication by others?

Yes

Reviewer Expertise:

Parent-child relationships, parental mental health, social and emotional development, cultural psychology

I confirm that I have read this submission and believe that I have an appropriate level of expertise to confirm that it is of an acceptable scientific standard, however I have significant reservations, as outlined above.

# Response

**Published:** 2022-01-11

**Authors:** Sunil Bhopal

This paper examines a Hindi version of a brief self-report measure of parental perceptions of their relationship (warmth, invasiveness) toward their 12-month-old infant in rural India. This version of the Mother-Object Relations Scale (MORS-SF) was utilised in a published clinical trial on preventing the impacts of early life stress (Bhopal et al., 2019), and this secondary paper describes how well the measure compares with 18-month developmental (e.g. cognitive, language, home environment quality) outcomes, as well as describes some of the psychometric characteristics of this translated version.

 The paper has potential high importance given what is known about how early parent-child relationships impact on various developmental outcomes, given how early stress that is very common in LMIC countries impacts on such relationships and given the ease of implementing this kind of measure in a low resource environment. The sample size obtained is very large for a study of this kind.

**We thank the reviewer for taking the time to read our paper in-depth and for these introductory remarks.**

 The introduction sets out the rationale very well and is accurate and impressively concise in presentation. While the MORS-SF has been available and in use for a while, the empirical literature seems not have caught up with that. For example, while the cited studies are of some relevance, the papers are: (1) a conference presentation – can details be provided for accessing the presentation poster or slides? (2) a Warwickshire (UK) study – while detailed, it applies to a different age group (2-4 years) and the number scales are modified for analysis; (3) a Hungarian study focused on postpartum depression and anxiety at 8-12 months postpartum; and (4) the aforementioned trial in which the MORS-SF is utilised as a screener with the current sample; those with moderate to high levels of concern were considered one of four relational risk factors (so no analysis details are provided specific to the MORS-SF). Therefore, while the MORS-SF is somewhat established and well-known, the psychometric data for the English language version for the relevant child age group is not as available or accessible as perhaps suggested. This clearly limits any ability to compare the findings here with other similar samples from different cultural contexts, including the UK where the measure originates. Perhaps further citations could be added to strengthen where possible, or there could be some acknowledgement in the limitations or as a general discussion point.

**We agree with the reviewer that the evidence-base supporting relatively widespread use of the MORS-SF tool continues to grow, and have added further relevant literature. We have now added the following which includes psychometric analysis of the English language version.**

**Oates** **, J. and Gervai, J. (2019) ‘Mothers’ perceptions of their infants’, Journal of Prenatal and Perinatal Psychology and Health, vol. 33(4) pp.282-300.**

**Oates** **, J., Gervai, J., Danis, I., Lakatos, K. and Davies, J. (2018) ‘Validation of the mothers’ object relations short-form (MORS-SF)’, Journal of Prenatal and Perinatal Psychology and Health, vol. 33(1) pp. 38–50.**

**And removed original reference 6 (validation study presented via conference poster)**

 A specific citation could be added to the assertion that mothers’ and fathers’ MORS-SF scores for the same child are not highly correlated.

**This is addressed in the 2018 validation paper and we have now specifically cited this finding in Introduction: Paragraph 2.**

**Oates** **, J., Gervai, J., Danis, I., Lakatos, K. and Davies, J. (2018) ‘Validation of the mothers’ object relations short-form (MORS-SF)’, Journal of Prenatal and Perinatal Psychology and Health, vol. 33(1) pp. 38–50.**

 The study design is appropriate, though it tries to be both a methodology and primary research paper. I applaud that the categories of concern as derived from the MORS-SF are provided here (Table 3) where they were not in Bhopal et al. (2019). The footnote of Table 3 states that the categories were defined prior to this study. It would be helpful to have clarification on how the categories of concern were defined and, if possible, a citation could be provided to support that.

**Thank you for this comment. This clarification is provided as requested in the paragraph immediately preceding Table 3: *‘* *MORS-SF defined ‘concern’ levels were calculated using a combination of these two sub-scale scores shown in* *Table 2* *and described in the footnote (these levels were described by Milford et al.* *^18^* *and not altered for this study)*’.**

 While there is certainly merit in studying the MORS-SF subscales in relation to these 18-month outcomes, it would also have seemed particularly appropriate (given the pre-validation stage of this translated version) to examine the MORS-SF in relation to the other 3 relational risk factors (mentioned in Bhopal et al., 2019) to give some indication of convergent validity. It would seem appropriate to discuss this somewhere in the discussion – perhaps as a limitation or as an important consideration for future research.

**Thank you. We have added this important consideration to the penultimate paragraph:**

**“Additional important future work will include examination of convergent validity with respect to other relational risk-factors, and further formal psychometric validation”.**

 The methods and analysis are detailed very well and are replicable. It would help the reader by offering Ns and % in the paragraph explaining MORS-SF responses (i.e. highlights of Figure 1).

**We have added these data to this paragraph as follows:**

**Figure 1** **shows considerable variation in responses to MORS-SF statements. In the warmth sub-scale the majority of mothers responded ‘nearly all the time’ to statements: “My baby… ‘smiles at me’ (826 mothers; 62.9%), ‘likes me’ (814; 62%), ‘laughs’ (754; 57.4%), and ‘likes to please me’ (758; 57.7%)”. These very positive ratings were less frequent for the statements: “My baby… ‘likes doing things with me’ (379; 28.9%), ‘talks to me’ (511; 38.9%) and ‘is affectionate towards me’ (648 (49.4%)”. In the invasion sub-scale, most mothers responded ‘never’ to the statements: “my child winds me up” (823; 62.7%) and “my child dominates me” (657; 50%); however, a considerable proportion indicated in response to the statements “my baby annoys me” and “my baby irritates me” that this happens rarely, sometimes or quite often (980; 74.6%, and 1005; 76.5% respectively). Many mothers responded to the statement “my baby wants too much attention” with ‘nearly all of the time’ (567; 43.2%).**

 The figures are interesting and generally well explained in the text. The detail of the translation process is very good yet concise, and the Hindi version of the measure is available for others to use. All source data is available.

**Thank you for these comments**

 The conclusions drawn are mostly adequately supported by the results, though this section could be strengthened by being a little more balanced in presentation and with the findings interpreted more critically.

 With regards to the former, there is a strong focus on the strengths of the study and the measure. I appreciate that one aim of the paper is surely to promote the viability of this brief measure, especially for use in LMIC contexts. Still, critical interpretation of the findings could be enriched and the limitations more thoroughly considered (ideas already mentioned above). One could unpack the different possible reasons for MORS-SF links with the later outcomes – for example, both variables could be linked to a third variable, such as socioeconomic deprivation. Were any variables controlled for?

**This point is well made. We have added to the limitations section and made light edits throughout the discussion section to attempt to address these comments and provide further balance to the section.**

 When considering translation, I appreciate that efforts went into ensuring that the meaning was maintained in the Hindi version. Given that this is a new translated version, I do wonder if there were still any concerns about some meaning being lost in translation. For example, many mothers agreed strongly that ‘my baby wants too much attention’ – it is possible that the connotations of this sentence could really differ across cultural contexts, with some interpretations being more negative towards the child than others might. Some reflection on translation issues could serve well for future usage and research, as well as translations into other languages, and for use in multicultural groups in clinical contexts. Considerations for the breadth of the audience will only serve to enhance what this paper already has to offer.

 Finally, thank you for the opportunity to review this interesting paper focused on an under-researched population with respect to parent-infant relationships and child outcomes.

**Thank you for the time you took to review our paper. We have added further detail on the translation issues.** **“The possibility of translations not being exact matches means that care should be taken, and further work done, if translated tools are to be used in multicountry (or multi-language in a single country) studies. We found the cognitive interviews and pre-testing to be of crucial importance and highly commend this approach to those conducting further work on the tool in varied languages.”**
